# Depression and risk of sarcopenia: a national cohort and Mendelian randomization study

**DOI:** 10.3389/fpsyt.2023.1263553

**Published:** 2023-10-18

**Authors:** Qian Zhong, Lisha Jiang, Kang An, Lin Zhang, Shuangqing Li, Zhenmei An

**Affiliations:** ^1^Department of Endocrinology and Metabolism, West China Hospital, Sichuan University, Chengdu, Sichuan, China; ^2^Day Surgery Center of West China Hospital, Sichuan University, Chengdu, Sichuan, China; ^3^General Practice Ward/International Medical Center Ward, General Practice Medical Center, National Clinical Research Center for Geriatrics, Multimorbidity Laboratory, West China Hospital, Sichuan University, Chengdu, Sichuan, China

**Keywords:** sarcopenia, depression, low muscle mass, CHARLS, older adults

## Abstract

**Background:**

Depression and the increased risk of sarcopenia are prevalent among the elderly population. However, the causal associations between these factors remain unclear. To investigate the potential association between depression and the risk of sarcopenia in older adults, this study was performed.

**Methods:**

In the baseline survey, a total of 14,258 individuals aged 40 and above from the China Health and Retirement Longitudinal Study (2015) participated. We initially described the baseline prevalence of the disease. Then, logistic regression and restricted cubic spline (RCS) regression were conducted to assess the relationship between depression and sarcopenia. Subgroup analysis was performed to validate the robustness of the findings. Additionally, we conducted Mendelian randomization analysis using the inverse variance weighting estimator to assess the causal relationship between depression and sarcopenia. Furthermore, we adopted six methods, including MR-Egger, simple median, weighted median, maximum likelihood, robust adjusted profile score (RAPS), and MR Pleiotropy Residual Sum and Outlier (MR-PRESSO), for sensitivity analyses.

**Results:**

Depression patients exhibited higher risks of sarcopenia in all five models adjusting for different covariates (*P* < 0.05). The RCS analysis demonstrated a linear relationship between depression and sarcopenia (*P* < 0.05). In the subgroup analysis, increased risk was observed among participants aged 60−70, married or cohabiting individuals, non-smokers, non-drinkers, those with less than 8 h of sleep, BMI below 24, and individuals with hypertension (all *P* < 0.05). Mendelian randomization results revealed that genetically proxied depression led to a reduction in appendicular skeletal muscle mass (all *P* < 0.05).

**Conclusion:**

Our study provides observational and causal evidences that depression can lead to sarcopenia. This finding emphasizes the importance of timely identification and management of depression, as well as implementing targeted educational programs as part of comprehensive strategies to prevent sarcopenia.

## Introduction

Currently, the global population is experiencing a growing trend of aging. In 2019, the global population of older adults was approximately 700 million (1). China, being the most populous country in the world, also has the largest elderly population. By the end of 2015, there were 143.9 million elderly adults in China, aged 65 years or older, comprising 10.5% of the total population (2). By the year 2050, it is projected that China will have a population of 400 million people aged 65 and above, with 150 million of them being 80 years old or older (3). The accelerated aging process also poses a substantial burden on social healthcare. It is predicted that the expenditure on pensions, healthcare, welfare, and facilities as a percentage of the gross domestic product (GDP) will increase from 7.33% in 2015 to 26.24% by 2050 (4). Among the various health-related factors that contribute to increased mortality in the elderly population, depression and sarcopenia have garnered significant attention.

Sarcopenia is a progressive and widespread age-related syndrome characterized by the reduction of skeletal muscle mass and strength, accompanied by decreased physical function (5). It typically develops slowly and covertly in older adults, particularly those aged 80 and above (6). Sarcopenia in older adults has been linked to functional impairments, disability, an elevated risk of falls and fractures, diminished health-related quality of life, and an increased mortality risk (7). Currently, there are no specific screening tools or diagnostic criteria for sarcopenia (8). However, assessing muscle mass, muscle strength, and daily activity capacity are important indicators for evaluating sarcopenia (9–11). The concept of sarcopenia was initially used to describe the age-related decline in skeletal muscle mass. In 1989, Irwin Rosenberg first used dual-energy X-ray absorptiometry (DXA) to measure and calculate ASMI [appendicular skeletal muscle mass (kg)/height (m^2^)]. ASMI below two standard deviations of healthy young individuals is diagnosed as low muscle mass. Although the diagnostic criteria for sarcopenia have been continuously updated, ASMI continues to be used as an important measure of muscle mass. This study adopted the diagnostic criteria for sarcopenia established by Irwin Rosenberg. Sarcopenia is a complex condition influenced by both environmental and genetic factors. Risk factors for sarcopenia include age, male gender, low BMI, malnutrition, poor lifestyle and long-term chronic disease history (12, 13). Additionally, there is evidence indicating that adverse psychological factors, such as depression, can contribute to an increased risk of sarcopenia.

Depression is a complex condition characterized by persistent feelings of low mood. It is typically pathological, long-lasting, and necessitates treatment (14). As the pressures of life increase, the prevalence of depression continues to escalate. In 2019, the China Mental Health Survey revealed a lifetime prevalence of depression at 6.9% and a 12°month prevalence of 3.6% (15). Elderly individuals are particularly vulnerable to experiencing long-term periods of low mood and various psychological disorders due to physical decline and decreased social adaptability. Depression is the most prevalent psychological disorder among the elderly (16). A recent meta-analysis demonstrated that the prevalence of depression among the elderly in China between 2010 and 2019 was 25.55% (17).

Sarcopenia and depression, as two common diseases in the elderly population, share some similarities in terms of clinical features, etiology, and prognosis, and they are bidirectionally related (18, 19). Sarcopenia may contribute to depression due to factors such as frequent falls, loss of independent living, disruptions in personal care, reduced nutritional intake, and lack of physical activity. Consequently, the prevalence of sarcopenia is higher in individuals with depression compared to the general elderly population (20). Similarly, symptoms associated with depression, such as weakness, loss of appetite, reluctance, and reduced motivation, can also contribute to the development of sarcopenia (19). Based on current evidence regarding risk factors for sarcopenia, the relationship between depression and sarcopenia has also been reported in previous studies. For example, Liu et al. (21) analyzed sarcopenia and its related factors using six different diagnostic criteria, including AWGS 2019, AWGS 2014, EWGSOP, EWGSOP2, IWGS, and FNIH. They found a significant association between depression and sarcopenia when using AWGS 2014 and FNIH criteria, while no significant correlation was observed when using the other four diagnostic criteria. Furthermore, Wu et al. (22) have recently highlighted an inverse correlation between depressive symptoms and muscle mass among elderly Chinese individuals. In support of this, a meta-analysis involving community-dwelling older adults has identified depression as an independent contributor to the development of sarcopenia (23). However, discrepancies in findings have emerged across various studies. Notably, the 2010-2011 Korean National Health and Nutrition Examination Survey yielded contrasting results, indicating no significant association between depression and sarcopenia.

Mendelian randomization (MR) serves as an epidemiological method that employs genetic variants to discern between correlation and causation in observational data. The credibility of an MR study hinges on the authenticity of the genetic variants utilized as instrumental variables (IVs) (24, 25). The core assumption underlying MR is that genetic variants are randomly allocated during conception and act as proxies for modifiable risk factors or exposures of interest. As such, they enable researchers to evaluate the causal effects of these factors on specific outcomes (26). Various methods can be employed to utilize genetic variants in MR analyses, each with its unique advantages and assumptions (27).

Therefore, considering the limited evidence in the Chinese population, the contradictory findings in current research, and the unclear causal relationship between depression and sarcopenia, this study utilized nationally representative data from the China Health and Retirement Longitudinal Study (CHARLS) in 2015 to investigate the observational relationship between depression and sarcopenia among Chinese middle-aged and elderly adults. The observational association was further extended to causal association using the MR method, addressing previous research limitations and informing interventions and prevention strategies.

## Materials and methods

### Data source and analytical sample

**FIGURE 1:**
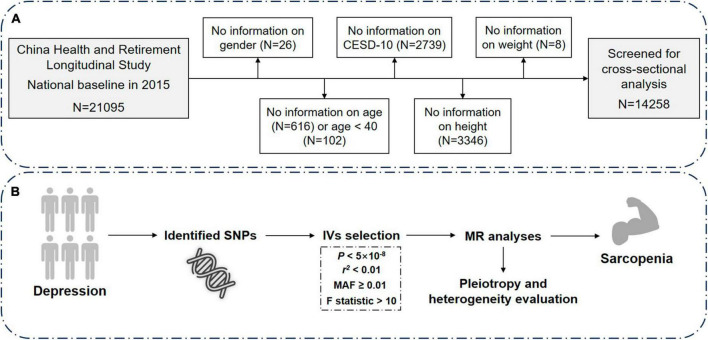
Overview of the study design and analysis strategy. **(A)** Schematic diagram of screening of the included research subjects. **(B)** Analysis strategy of MR. Filtered SNPs that meet the criteria are considered as instrumental variables (IVs) and undergo sensitivity analyses, heterogeneity and pleiotropy evaluation. Potential biases from sample overlap and statistical power are also assessed. The results from both the discovery and replication stages are then combined. N, number; CESD-10, Center for Epidemiologic Studies Depression Scale-10; MR, Mendelian randomization; SNP, single nucleotide polymorphism.

The cross-sectional data for this study were obtained from CHARLS, a nationwide longitudinal survey that utilizes the multi-stage probability-proportional-to-size sampling method to select residents aged 45 and above from 28 provinces, 150 counties, and 450 villages in China for tracking and follow-up. CHARLS covers various information related to households, health, and healthcare and serves as a high-quality database reflecting the health of the middle-aged and elderly population in China (28). The baseline survey was conducted in 2011, with a total of 17,708 participants. Follow-up surveys are conducted every 2°years, and currently, there are four waves of data available (2011, 2013, 2015, and 2018). However, only two waves (2011 and 2015) had blood samples. For this study, data from the third wave of the survey conducted in 2015 were utilized, which contained indices from blood like creatinine and cystatin C. The sample size in this wave was 21,095. After excluding individuals with missing information on gender, age, depression, sarcopenia, or age less than 40 years, the cross-sectional analysis included 14,258 participants (Figure 1A).

### Assessment of depression and sarcopenia

The assessment of depressive symptoms in middle-aged and elderly individuals employed the Center for Epidemiological Studies Depression Scale-10 (CESD-10). The Chinese version of CESD has been extensively used among the elderly population in China and has demonstrated robust reliability and validity (29). CESD focuses on the individual’s experiences during the past week and classifies symptom frequency into four categories: “rarely or none,” “occasionally,” “frequently,” and “almost always.” Each category is assigned a score of 0, 1, 2, or 3, respectively. The total score ranges from 0 to 30, with higher scores indicating more severe depressive symptoms. In this study, scores ≥10 were diagnosed as depression, in line with previous studies (30, 31).

Sarcopenia was assessed based on the criteria recommended by the Asian Working Group for Sarcopenia (AWGS) in 2019, which includes measures of muscle strength, appendicular skeletal muscle mass (ASM), and physical performance (9). The presence of abnormal muscle mass can lead to a diagnosis of sarcopenia. Muscle mass was estimated using the previously validated anthropometric equation for Chinese individuals (32, 33). The following equation for height-adjusted muscle mass (ASM/Ht^2^) can be used to determine the presence of low ASM in the Chinese population (32): ASM/Ht^2^ = [0.193 × weight (kg) + 0.107 × height (cm)°−°4.157 × gender°−°0.037 × age (years)°−°2.631]/height^2^. Several studies have shown strong consistency between the ASM equation model and dual-energy X-ray absorptiometry (DXA) measurements (32, 33). The threshold for defining low muscle mass is based on the lowest 20% of gender-specific height-adjusted muscle mass (ASM/Ht^2^) in the study population (34). Therefore, an ASM/Ht^2^ value <5.42 kg/m^2^ for females and <7.08 kg/m^2^ for males is considered indicative of low muscle mass.

### Potential covariates

Based on previous studies, we collected several potential confounding variables to adjust for the association between depression and skeletal muscle mass (4, 35). These variables include individual sociodemographic characteristics and health-related factors. Sociodemographic variables include age, gender, and marital status. Health-related factors include smoking, alcohol consumption, daily sleep duration, afternoon napping, body mass index (BMI), hypertension, uric acid, low-density lipoprotein (LDL), total cholesterol (TC), blood urea nitrogen (BUN), creatinine, cystatin C, and creatinine-to-cystatin C ratio (CCR).

Age was categorized into four subgroups: 40−50, 50−60, 60−70, and ≥70 years. Marital status was stratified as married and living together/cohabitated or separated/divorced/widowed/never married/married but not living together. BMI was divided into four groups: underweight (<18.5 kg/m^2^), normal weight (18.5 kg/m^2^ ≤ BMI < 24.0 kg/m^2^), overweight (24.0 kg/m^2^ ≤ BMI < 28.0 kg/m^2^), and obese (BMI ≥ 28.0 kg/m^2^) (36). Sleep duration was categorized as <6 h, 6−8 h, and >8 h. Afternoon napping was classified as yes or no. Smoking status included current smoker, non-smoker, and ex-smoker. Alcohol consumption was classified as once a month, less than once a month, and never. Hypertension was defined as systolic pressure ≥140 mmHg or diastolic pressure ≥90 mmHg according to the World Health Organization (WHO) definition. If someone has a history of hypertension and is on medication, but their blood pressure stays below 140/90 mmHg, they are still classified as having hypertension (37). Hyperuricemia was defined as a serum uric acid level ≥420°μmol/L in men or ≥357°μmol/L in women (38). According to the WHO cardiovascular disease (CVD) risk chart, the indication to start statin therapy was considered if the patient was classified as high risk, moderate risk if >40 years with TC > 200 mg/dL or LDL-C > 120 mg/dL (39). Additionally, previous studies have indicated that CCR can serve as a serum biomarker to predict muscle mass, and this ratio has been shown to be positively correlated with muscle mass (40).

### Data sources of depression and appendicular skeletal muscle mass

The genetic estimates of major depression were retrieved from the Major Depressive Disorder Working Group of the Psychiatric Genomics Consortium (41). A total of 5,00,199 individuals (1,70,756 cases and 3,29,443 controls) from European descent were included. The diagnosis of depression was based on self-report in online surveys and clinical diagnosis in medical histories. Detailed information regarding the quality control, phenotype definition, and statistical analyses can be accessed in the original study by the Psychiatric Genomics Consortium. The genetic associations of appendicular skeletal muscle mass were obtained from one previous study by Pei et al. (42). This study included 4,50,243 individuals from UK Biobank. Appendicular skeletal muscle mass was quantified by the sum of fat-free mass at the arms and legs. All the participants were from the White descent.

### Selection of instrumental variables in MR analyses

The IVs were retrieved at a genome-wide statistical significance of *P* < 5 × 10^–8^. To obtain the independent IVs, SNPs with linkage disequilibrium *r*^2^ ≥ 0.01 at a window size of 10,000 kb were excluded. Additionally, SNPs with minor allele frequency (MAF) <0.01 and palindromic SNPs were also excluded. No proxy SNPs were used as IVs in MR analyses (Figure 1B). Finally, 43 SNPs were qualified as IVs, showed in Supplementary Table 1.

### Statistical analysis in CHARLS

In this study, continuous variables with a normal distribution were presented as mean ± standard deviation (SD), while continuous variables with a non-normal distribution were presented as median (25–75th percentile). Student’s *t*-test for continuous data and Chi-square test for categorical data were used to assess the differences in baseline characteristics. A set of logistic regression models was built to investigate the relationship between depression and sarcopenia. Five models were utilized with different combinations of covariates. Specifically, Model 1 was the crude model, Model 2 included age, gender and marital status, Model 3 additionally included sleep duration and afternoon napping, Model 4 further included smoking and alcohol consumption, and Model 5 then further adjusted for BMI, hypertension, uric acid, LDL and TC. Odds ratios (ORs) and 95% confidence intervals (CIs) were calculated based on the logistic regression models. Moreover, we also used the restricted cubic spline (RCS) regression (43) to investigate the non-linear association between depression and sarcopenia.

### Statistical analysis in MR

To assess the causal relationship between depression and appendicular skeletal muscle mass, MR was performed. The IVW (44) method was used as the main analysis due to the high statistical power for valid IVs. This method uses a meta-technique to combine the Wald ratio of IVs and then yield the causal estimates. In addition, we also adopted six methods for sensitivity analyses including MR-Egger, simple median, weighted median, maximum likelihood, robust adjusted profile score (RAPS), and MR Pleiotropy Residual Sum and Outlier (MR-PRESSO). The MR-Egger and weighted median approaches can produce consistent results when invalid IVs exist. MR-Egger (45) is adapted from Egger regression and introduces an intercept term in the regression model. The distance between the null and intercept term was used to detect the horizontal pleiotropy. Weighted median estimator can produce robust results even when 50% IVs are invalid. The maximum likelihood method has advantage in limited sample size, which displays minimal bias. For MR.RAPS (46), it can yield consistent causal estimates when weak and pleiotropic IVs exist. The MR-PRESSO (47) considers the pleiotropic outliers and can combine the Wald ratio as IVW method after excluding pleiotropic IVs. Additionally, we also employed Cochran *Q* test quantify the heterogeneity. To minimize the bias from weak instrumental variable (IV), we also assessed the F statistics using the following equation: F statistics = (Beta/Se)^2^ (48).

A two-tailed *P*-value <0.05 was considered statistically significant. All statistical analyses and figures were made using STATA software (version 17.0, Stata Corp, College Station, TX, USA) or R 4.0.3 (R Foundation for Statistical Computing, Vienna, Austria).

## Results

### Baseline characteristics of the participants in 2015

A total of 14,258 individuals participated in the baseline survey of CHARLS in 2015 (Table 1). The study population was divided into two groups: the non-sarcopenia group with 11,407 participants and the sarcopenia group with 2,851 participants. Except for gender (*P* = 0.98), all covariates in Table 1 were statistically imbalanced (*P* < 0.05). Compared to individuals without sarcopenia, those with sarcopenia were older, had a higher proportion of unfavorable marital status, lower BMI, shorter daily sleep duration, lower afternoon nap rate, lower levels of uric acid, LDL, and TC, and higher prevalence of smoking, hypertension, and BUN levels.

**TABLE 1:** Characteristics of the participants attending the baseline survey.

Characteristics	Non-sarcopenia *N* = 11,407	Sarcopenia *N* = 2,851	*P*-value
Age (years)	57.62 (9.18)	65.73 (9.85)	<0.001
Gender			
Male	5,435 (47.6%)	1,359 (47.7%)	0.98
Female	5,972 (52.4%)	1,492 (52.3%)	
Marital status			<0.001
Married/cohabitating	9,746 (85.4%)	2,192 (76.9%)	
Others	1,661 (14.6%)	659 (23.1%)	
Sleep duration (hours)			<0.001
0−6 h	5,501 (48.5%)	1,536 (54.6%)	
6−8 h	4,799 (42.4%)	974 (34.6%)	
>8 h	1,031 (9.1%)	303 (10.8%)	
Afternoon napping			<0.001
No	4,603 (40.5%)	1,345 (47.4%)	
Yes	6,766 (59.5%)	1,493 (52.6%)	
Smoking			<0.001
Current smoker	3,072 (26.9%)	971 (34.1%)	
Non-smoker	6,846 (60.0%)	1,543 (54.1%)	
Ex-smoker	1,485 (13.0%)	336 (11.8%)	
Alcohol consumption			<0.001
More than once a month	3,159 (27.7%)	754 (26.5%)	
Less than once a month	1,061 (9.3%)	201 (7.1%)	
Never	7,182 (63.0%)	1,894 (66.5%)	
BMI (kg/m^2^)			<0.001
<18.5	1 (0.0%)	755 (26.5%)	
18.5−24	4,730 (41.5%)	2,078 (72.9%)	
24−28	4,737 (41.5%)	15 (0.5%)	
≥28	1,939 (17.0%)	3 (0.1%)	
Hypertension			<0.001
No	6,870 (60.7%)	1,982 (70.2%)	
Yes	4,451 (39.3%)	842 (29.8%)	
Uric acid (mg/dL)			<0.001
Non-hyperuricemia	8,239 (87.6%)	2,078 (91.9%)	
Hyperuricemia	1,165 (12.4%)	183 (8.1%)	
LDL			<0.001
≤120 mg/dL	7,035 (75.0%)	1,798 (79.7%)	
>120 mg/dL	2,342 (25.0%)	457 (20.3%)	
TC			0.007
≤200 mg/dL	6,587 (70.2%)	1,649 (73.1%)	
>200 mg/dL	2,791 (29.8%)	606 (26.9%)	
BUN			<0.001
<20 mg/dL	8,167 (87.1%)	1,820 (80.7%)	
>20 mg/dL	1,211 (12.9%)	435 (19.3%)	
Creatinine (mg/dL)	0.80 (0.30)	0.81 (0.26)	0.044
Cystatin C (mg/L)	0.83 (0.23)	0.89 (0.24)	<0.001
CCR	0.98 (0.27)	0.93 (0.25)	<0.001

BMI, body mass index; LDL, low-density lipoprotein; TC, total cholesterol; BUN, blood urea nitrogen; CCR, creatinine-to-cystatin C ratio.

### The association between depression and sarcopenia

To assess the association between depression and sarcopenia, five models were constructed (Table 2). As a binary variable, the crude model showed that the depressive group had a 1.49-fold higher risk of sarcopenia compared to the non-depression group [95% confidence interval (CI): 1.37−1.62, *P* < 0.001]. The results remained consistent in different models [odds ratios (ORs) = 1.41 for Model 2, 1.37 for Model 3, 1.36 for Model 4, and 1.24 for Model 5, all *P* < 0.05]. As a continuous variable, all the five models supported the positive association between CESD-10 questionnaire scores and sarcopenia (ORs = 1.04 for the crude model, 1.03 for Model 2, Model 3, and Model 4, and 1.02 for Model 5, all *P* < 0.001).

**TABLE 2:** Logistic regression models on depression score and sarcopenia.

Models	Depression [ORs (95% CI)]	*P*-values	CESD-10 scores [ORs (95% CI)]	*P*-values
Model 1	1.49 (1.37−1.62)	<0.001	1.04 (1.03−1.04)	<0.001
Model 2	1.41 (1.28−1.54)	<0.001	1.03 (1.02−1.04)	<0.001
Model 3	1.37 (1.25−1.51)	<0.001	1.03 (1.02−1.03)	<0.001
Model 4	1.36 (1.24−1.49)	<0.001	1.03 (1.02−1.03)	<0.001
Model 5	1.24 (1.08−1.42)	0.002	1.02 (1.01−1.03)	<0.001

Depression is a binary variable, defined as CESD-10 scores ≥10; CESD-10, Center for Epidemiologic Studies Depression Scale-10; OR, odds ratios; CI, confidence interval. Model 1: Crude model. Model 2: Adjusted for age, gender and marital status. Model 3: model 2 + sleep duration and afternoon napping. Model 4: model 3 + smoking and alcohol consumption. Model 5: model 4 + BMI, hypertension, uric acid, LDL and TC.

### RCS regression between depression and sarcopenia

**FIGURE 2:**
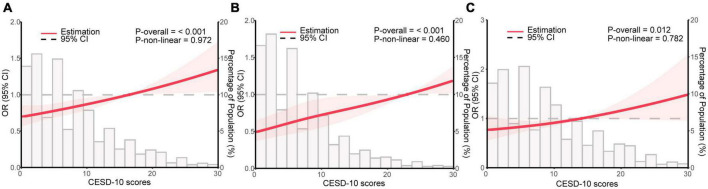
Restricted cubic spline regression analysis for depression and sarcopenia. The red area is plotted using data from the 95% CI. **(A)** General population; **(B)** male; **(C)** female. OR, odds ratio; CI, confidence interval; CESD-10, Center for Epidemiological Studies Depression Scale-10.

**FIGURE 3:**
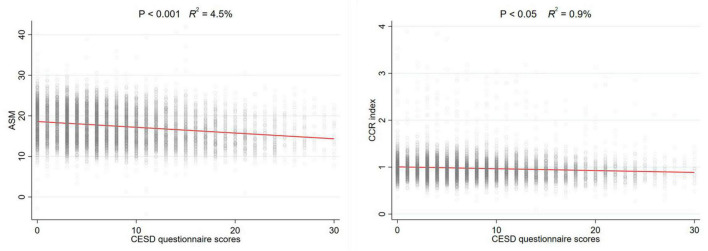
The linear relationship between CESD-10 scores, ASM, and CCR. ASM, appendicular skeletal muscle mass; CCR, creatinine-to-cystatin C ratio.

To investigate the non-linear relationship between CESD-10 scores and risk of sarcopenia, RCS regression was performed. As shown in Figure 2A, there was a linear dose-response relationship between depression and sarcopenia (*P* for overall <0.001) in the overall population. Furthermore, we performed the RCS regression in males and females, respectively. Similarly, there were no non-linear associations between CESD-10 scores and risk of sarcopenia in males (*P* for overall <0.001, Figure 2B) or females (*P* for overall <0.05, Figure 2C). It was revealed that with the increase of CESD-10 scores, the risk of developing sarcopenia continuously elevated. In Figure 3, we fitted the linear relationship between CESD-10 scores, ASM, and CCR. The results indicated that higher CESD-10 scores were linearly correlated with lower ASM and CCR (both *P* < 0.05).

### Subgroup analyses

**FIGURE 4:**
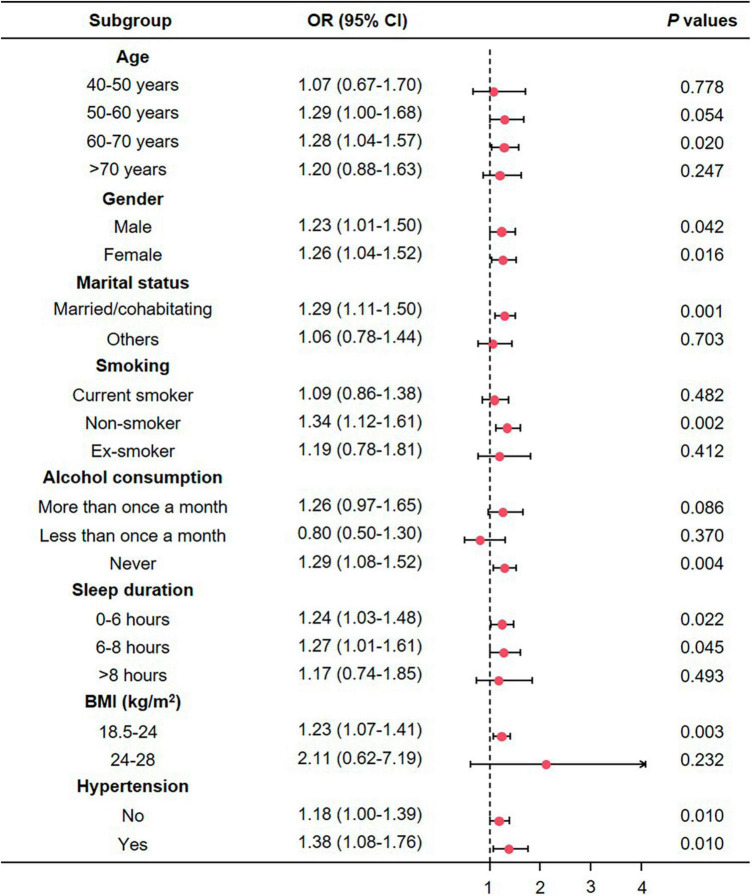
The association between depression and sarcopenia in subgroups. CI, confidence interval; OR, odds ratio; BMI, body mass index.

To further investigate the potential subgroup-specific relationship between sarcopenia and depression, we conducted subgroup analyses using univariate logistic regression models (Figure 4). No significantly increased risks were found among participants aged <60 or >71 (*P* > 0.05), those who were not married or cohabitating (*P* = 0.703), current or former smokers (*P* > 0.05), individuals who drank more or less than once a month (*P* > 0.05), participants who slept more than 8 h (*P* = 0.493), and those with a BMI in the overweight range (*P* = 0.232). However, we observed elevated risks in the 61−70 age group (*P* < 0.05), across all gender groups (*P* < 0.05), among participants who were married or cohabitating (*P* < 0.001), non-smokers (*P* < 0.05), non-drinkers (*P* < 0.05), individuals who slept less than 8 h (*P* < 0.05), those with a normal BMI (18.5−24 kg/m^2^, *P* < 0.05), and participants with and without hypertension (*P* < 0.05).

### Genetically proxied depression reduced appendicular skeletal muscle mass

**FIGURE 5:**
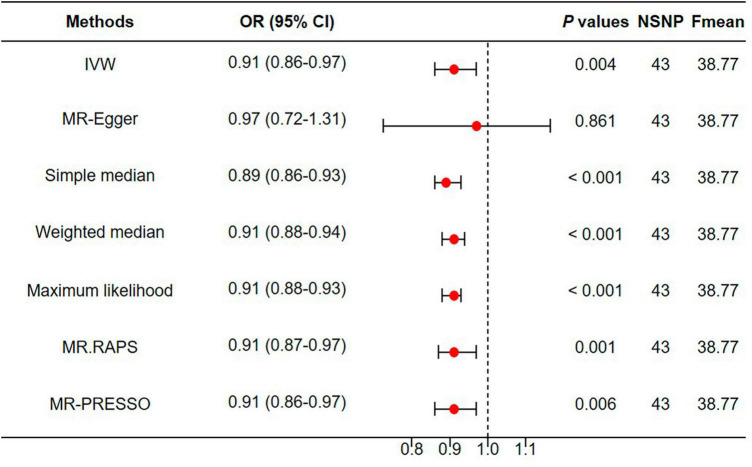
Genetically proxied depression reduced appendicular skeletal muscle mass. MR, Mendelian randomization; NSNP, number of single nucleotide polymorphisms; OR, odds ratio; CI, confidence interval.

**FIGURE 6:**
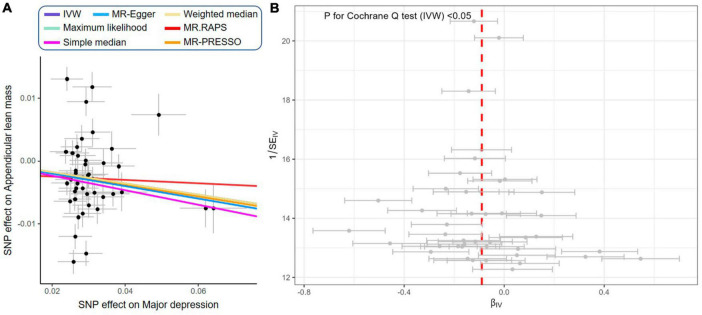
Scatter plot and funnel plot of genetically proxied depression on reduced appendicular skeletal muscle mass. **(A)** Scatter plot showing the effects of SNPs on major depression and appendicular skeletal muscle mass: as the SNP effect on depression increases, the SNP effect on appendicular skeletal muscle decreases. IVW, MR-Egger, weighted median, maximum likelihood, simple median, MR.RAPS, and MR-PRESSO represent results from these regression analyses. **(B)** Funnel plot showing the overall heterogeneity of MR estimates for the effect of major depression on appendicular skeletal muscle mass.

As shown in Figure 5, the IVW estimator revealed that genetically proxied depression reduced appendicular skeletal muscle mass (OR = 0.91, 95% CI = 0.86−0.97, *P* = 0.004). Five other methods reported similar estimates (OR = 0.89 for simple median, 0.91 for weighted median, maximum likelihood, MR.RAPS, and MR-PRESSO, all *P* < 0.01). The result from MR-Egger was insignificant (*P* = 0.861), however, the effect size and direction were accordant (OR = 0.97, 95% CI = 0.72−1.31). In Figure 6A, it was observed that with the SNP effect on major depression, the SNP effect on appendicular skeletal muscle mass decreased. The funnel plot detected the presence of heterogeneity in Figure 6B (*P* < 0.05). Therefore, the random-effect IVW model was used in our MR analyses (*P* = 0.004).

## Discussion

Our study findings indicate an increased risk of sarcopenia associated with depression, with higher CESD-10 scores being correlated with lower reported ASM values. This is consistent with existing cross-sectional evidence on the correlation between depression and sarcopenia and its components, despite the use of different measures to assess depression (35, 49–53). In an early observational study by Yanagita et al. (54), which included 2,856 Japanese-American men aged 71−93 years, depressed individuals showed a significant decline in gait speed compared to the healthy population. Subsequently, Kim et al. (53) found that among elderly male participants with depression, the mean ASM value was significantly lower compared to non-depressed individuals. In a recent observational study conducted in the United States, involving 3,421 older adults, it was discovered that individuals with depression exhibited significantly lower handgrip strength, particularly among females (55). A meta-analysis of 11 studies revealed that depression was an independent factor for sarcopenia (23). Kurita et al. (56) conducted a longitudinal analysis over a period of 1°year in patients with advanced chronic kidney disease, revealing a significant association between depression and an increased risk of developing sarcopenia. However, the limited number of specific individuals and the relatively short follow-up time restricted the ability to evaluate the causal relationship between depression and sarcopenia. In our study, after adjusting for potential confounding factors, we found that compared to the non-depressed group, individuals with depression had a 24% increased risk of incident sarcopenia. Importantly, we utilized Mendelian randomization to investigate the causal link between depression and sarcopenia. This method hinges on identifying genetic variants associated with these conditions and subjecting them to rigorous statistical analysis, using a stringent significance threshold to confirm robust associations (48). The IVs are qualified with clear biological explanation linking variants to phenotypes. For example, SNP rs1021363 is situated within the intron of the *SORCS3* (Sortilin-related vacuolar protein sorting 10 domain containing receptor 3) gene. *SORCS3* gene expression is exclusive to the nervous system, predominantly associated with vesicular components. Notably, neuronal activity triggers the expression of *SORCS3* in the hippocampus (57). Furthermore, *SORCS3* plays a role in modulating both NMDA receptor-dependent and -independent long-term depression, thereby serving as a postsynaptic regulator of synaptic depression and fear extension (57). SNP rs10235664 is situated within the intron of the *MAD1L1* (Mitotic arrest deficient 1 like 1) gene. *MAD1L1* serves as a crucial component of the mitotic spindle assembly checkpoint, primarily localized on kinetochores. It is noteworthy that previous studies have identified numerous genetic variants within *MAD1L1* that have been linked to psychiatric disorders (58). The qualified IVs provide more reliability to our findings. In summary, these results suggest that assessing depression in older adults may facilitate the identification of those at the highest risk of developing incident sarcopenia, who would benefit most from early intervention.

It is currently believed that the interaction between sarcopenia and depression may be attributed to shared underlying factors, primarily including physical inactivity, nutritional factors, chronic inflammation, and hormonal imbalance (18, 59). Individuals with depression often experience reduced physical activity and decreased dietary intake, which are recognized as contributing factors to the development of sarcopenia (18). Furthermore, inflammation is a common mechanism linking sarcopenia and depression. Age-related chronic low-grade inflammation characterized by elevated levels of IL-6 and TNF-α has been reported as an important factor in the occurrence of sarcopenia and the development of depression (60, 61). Studies have also shown that decreased levels of vitamin D contribute to the onset of sarcopenia (62), while deficiencies in vitamin D increase the risk of depression (63). As sarcopenia is often associated with loss of physical independence, frequent falls, and poor quality of life, these conditions may also lead to the occurrence of depression (64). Therefore, sarcopenia and depression share many common risk factors and pathophysiological mechanisms, which contribute to their bidirectional relationship. However, research on the causal relationship and underlying mechanisms between sarcopenia and depression remains limited.

Subgroup analyses provide valuable insights by helping clinicians understand how study findings are applicable to specific patient populations (65). In terms of age, only individuals aged 60−70 years had statistical significance, indicating that younger age may have a protective effect. Early study found that skeletal muscle mass began to decline from around 25 years old and thereafter accelerates (66). Typically, the projected prevalence of sarcopenia among the elderly, particularly individuals aged 60−70 years, ranges from 5 to 13%. This prevalence escalates significantly to a range of 11–50% among those aged over 80 years (67). However, our subgroup analyses did not yield statistically significant results for the population aged 70 and above, which could potentially be attributed to a limited sample size. In terms of sleep duration, patients with shorter sleep duration have a higher risk of developing sarcopenia. Generally, longer sleep duration indicates higher sleep quality and lower risk of sleep-related disorders, which is closely related to depression (68). On the other hand, individuals with depression are inherently sleep-deprived (69). Therefore, individuals participating in this experiment who lack sleep have a higher risk of depression compared to those who rest well, leading to an increased incidence of sarcopenia.

However, surprisingly, the study found that individuals who never drink alcohol have an increased risk of developing sarcopenia, which may reveal a potential protective effect of moderate alcohol consumption. Previous studies aimed at evaluating this phenomenon have also yielded different results with no unified conclusion. Skinnerd et al. (70) conducted a cross-sectional analysis on 1,96,561 white participants from the UK Biobank and performed a longitudinal analysis on a subset of 12,298 participants. The findings revealed a peak at medium levels of alcohol consumption, followed by a significant decline as alcohol consumption increased in the cross-sectional analysis. However, the longitudinal study results indicated no association between alcohol consumption and muscle mass. Another intriguing finding is that individuals with a favorable marital status have a higher risk of developing sarcopenia compared to those with an unfavorable marital status. This could be attributed to the fact that individuals with a favorable marital status might be more inclined to consume diets high in calories and fats or exhibit irregular eating habits due to increased social interactions and activities (71). These behaviors could result in sarcopenia (72). Additionally, individuals with a favorable marital status might lead sedentary lifestyles or lack regular physical exercise due to the time demands linked to their marital roles (73), thereby increasing the risk of muscle loss (74). However, in terms of baseline characteristics, individuals diagnosed with sarcopenia were characterized by a higher proportion of unfavorable marital status. Consistent with existing research, most current studies indicate that sarcopenia tends to be associated with lower educational levels and the absence of a spouse, as opposed to individuals without sarcopenia (75, 76). Consequently, while a favorable marital status is generally associated with a more stable social environment and support network, specific cases might present lifestyle factors unfavorable to muscle health, potentially elevating the risk of sarcopenia. Further research is required to explore the mechanisms and factors underlying this phenomenon. Participants with a BMI < 24 kg/m^2^ were observed to have an increased risk, consistent with previous research indicating that lower BMI is a risk factor for sarcopenia (77). Furthermore, individuals with hypertension had a higher risk of developing sarcopenia compared to those without hypertension. Gender, on the other hand, had minimal impact on the risk of sarcopenia.

This study has several advantages. First, we utilized a large nationwide representative sample, which provides our research findings with extensive generalizability to the elderly population in China. Second, unlike previous cross-sectional studies, this is the first study to employ Mendelian randomization to explore the causal relationship between depression and sarcopenia.

However, it is important to acknowledge the limitations of this study. Firstly, the use of observational data introduces potential bias and confounding factors. While we attempted to account for as many relevant factors as possible in our analysis, there may still be unmeasured confounders such as nutritional status, hypoalbuminemia, dietary habits, and physical activity. Additionally, the reliance on self-reported data introduces the possibility of recall bias, which is inherent in questionnaire surveys. Secondly, the use of an anthropometric equation instead of DXA or BIA to assess ASM. However, this equation has been validated in the Chinese population (32). Our threshold for low ASM aligns with previous studies conducted by Hu et al. (4, 78). Furthermore, using an anthropometric equation for assessing low ASM may offer a cost-effective alternative to BIA or DXA, especially in large-sample population-based studies (79). Additionally, the variability in the definition of depression and sarcopenia across different studies limits the comparability of results (80). Future research should strive for consistent definitions or diagnostic criteria for depression and sarcopenia to confirm their relationship. Thirdly, although Mendelian randomization studies indicate a stronger correlation between depression and sarcopenia compared to cross-sectional studies, the underlying biological mechanisms remain unclear. Further experimental research is necessary to establish and understand this association. Fourthly, the current approach to examining the relationship between depression and sarcopenia often treats the presence of depression as a binary variable. However, this approach overlooks the specific symptom items and treats all data as equivalent and interchangeable indicators of depression (81). Moreover, individuals with the same CESD-10 score may exhibit varying clinical severity of symptoms in relation to sarcopenia. To date, no studies have investigated the relationship between specific depressive symptomatology, as measured by CESD-10, and sarcopenia status in the Chinese elderly population. In our study, we acknowledge that the approach we have adopted involves diagnosing depression through self-reported assessments. However, when it comes to larger-scale epidemiological investigations, relying solely on clinical methodologies for depression diagnosis can prove to be a challenge due to the extensive scope of implementation. Therefore, we made the deliberate choice to employ a self-report questionnaire, a decision that has gained widespread acceptance in extensive epidemiological research and has also been employed in prior studies. Lastly, the gap from depression in CHARLS and major depression in MR is also a limitation in our study. Although the RCS regression also supported the linear correlation between severity of depression and ALM, there was no cut-off to define major depression in CHARLS. Further studies should further explore their association in patients with major depression.

## Conclusion

In conclusion, our study provides observational and causal evidences that depression can lead to sarcopenia. This finding emphasizes the importance of timely identification and management of depression, as well as implementing targeted educational programs as part of comprehensive strategies to prevent sarcopenia.

## Data availability statement

Publicly available datasets were analyzed in this study. This data can be found here: http://charls.pku.edu.cn/ and https://gwas.mrcieu.ac.uk/.

## Ethics statement

The studies involving humans were approved by the Ethics Committee of Peking University. The studies were conducted in accordance with the local legislation and institutional requirements. The participants provided their written informed consent to participate in this study.

## Author contributions

QZ: Formal analysis, Methodology, Software, Writing – original draft. LJ: Data curation, Formal analysis, Resources, Software, Writing – original draft. KA: Conceptualization, Methodology, Supervision, Visualization, Writing – original draft. LZ: Data curation, Investigation, Methodology, Writing – review and editing. SL: Funding acquisition, Project administration, Supervision, Validation, Visualization, Writing – review and editing. ZA: Project administration, Resources, Software, Supervision, Writing – review and editing.
